# Supplementary material for: Shared and unique consequences of Joubert Syndrome gene dysfunction on the zebrafish central nervous system
Source: Biol Open. 2024 Nov 12;13(11):bio060421. doi: 10.1242/bio.060421 (PMC11583916; doi:10.1242/bio.060421)
Supplement: Supplementary information [file biolopen-13-060421-s1.pdf]

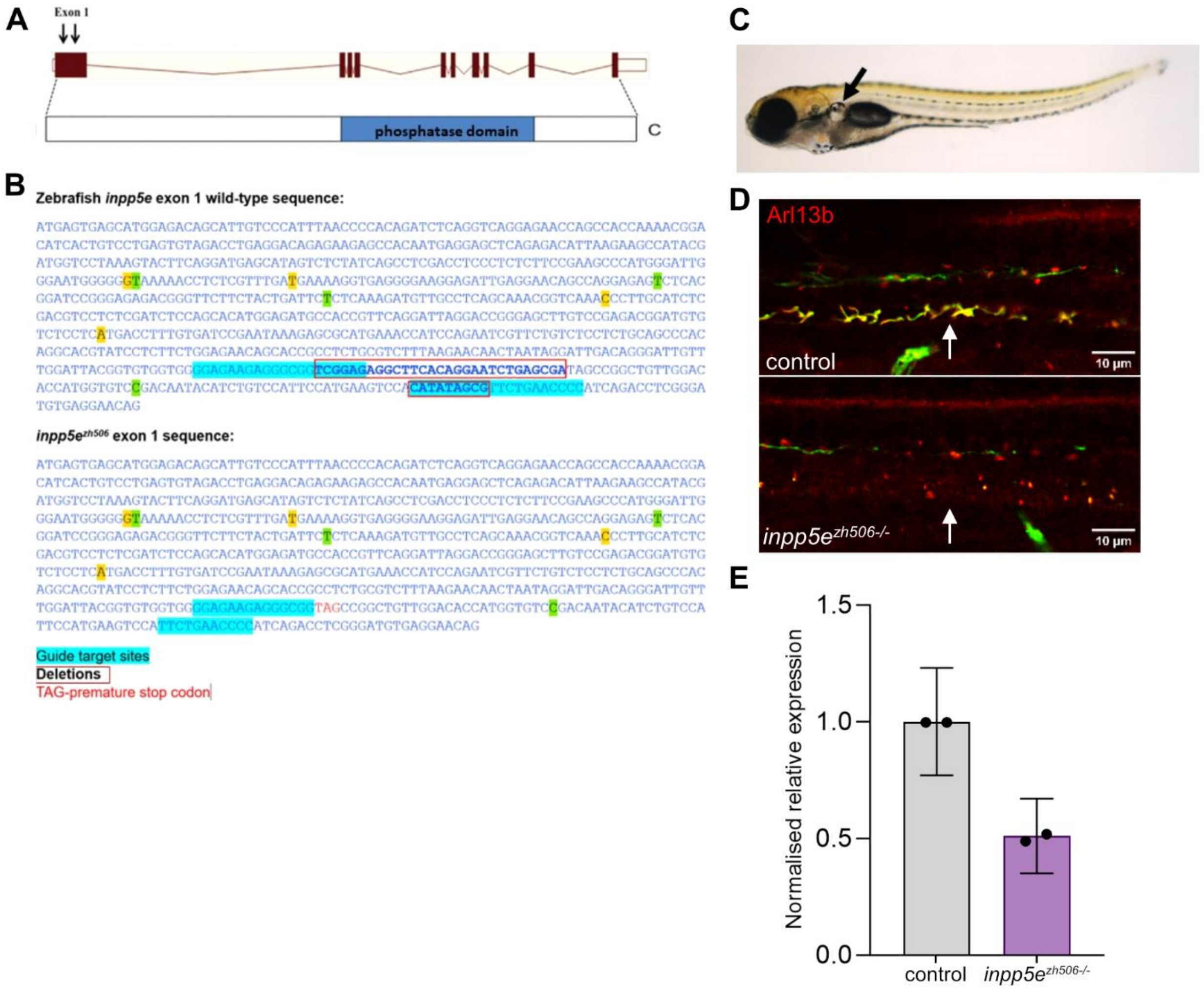

Fig. S1. Generation and validation of *inpp5e*^{zh506} mutant

(A) Schematic of the zebrafish *inpp5e* gene and protein indicating the CRISPR target sites in exon 1, the exons as brown bars in the gene schematic on top and the phosphatase domain in blue in the protein schematic on the bottom. **(B)** Sequence of zebrafish *inpp5e* exon 1 in wild-type zebrafish (top) and in the *inpp5e*^{zh506} mutant (bottom). The sgRNA target sites are marked in turquoise, the deleted regions are bolded and boxed in red and the first STOP codon (TAG) encountered after the deletion is labelled in red. SNPs according to ensembl annotation are marked in green or yellow (synonymous or missense, respectively). **(C)** 5 dpf old *inpp5e*^{zh506} mutant larva displaying mild body curvature and kidney cyst (arrow). **(D)** Immunofluorescence image with anti-Arl13b (Arl13b – red) and anti-acetylated tubulin (AcTub – green) showing cilia in the pronephric duct (arrow) in control (top) and mz *inpp5e*^{zh506} mutant (bottom, n = 10, N = 2 independent experiments). Note the strong decrease in cilia numbers and length in the mutant. Scale bar is 10 µm. **(E)** qRT-PCR analysis showing that normalised relative expression of *inpp5e* is decreased in *inpp5e*^{zh506} mz mutants compared to controls at 72 hpf. Data points represent samples from independent experiments (i.e. larvae from independent clutches).

Biology Open • Supplementary information

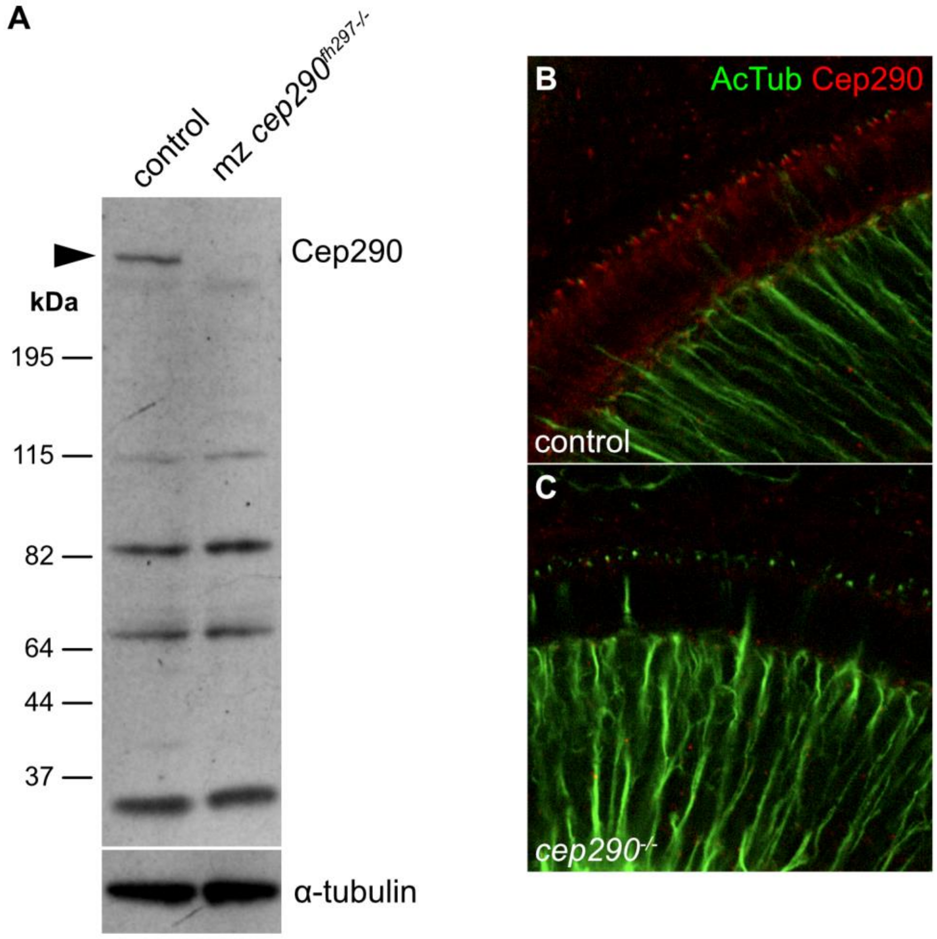

Fig. S2. ***cep290^{fh297}*** **mutants show loss of full-length Cep290 protein**

(A) Western blot with antibodies against the C terminus of Cep290 (top) and α-tubulin (bottom) performed on whole control and mz *cep290^{fh297}*-/- embryo lysates at 2 dpf. The Cep290 band (black arrowhead) appears in controls as >195 kDa and is virtually absent in mutants. α-tubulin is used as a loading control. **(B-C)** Whole-mount confocal immunofluorescence images showing a portion of the retina in control (B) and mz *cep290^{fh297}*-/- larvae at 3 dpf, stained with antibodies against AcTub (AcTub – green) and the C terminus of Cep290 (Cep290 – red) (n = 5 control and n = 5 *cep290* mutant larvae, N = 1 independent experiment). Note the virtual absence of Cep290 signal in mutants compared to controls.

Biology Open • Supplementary information

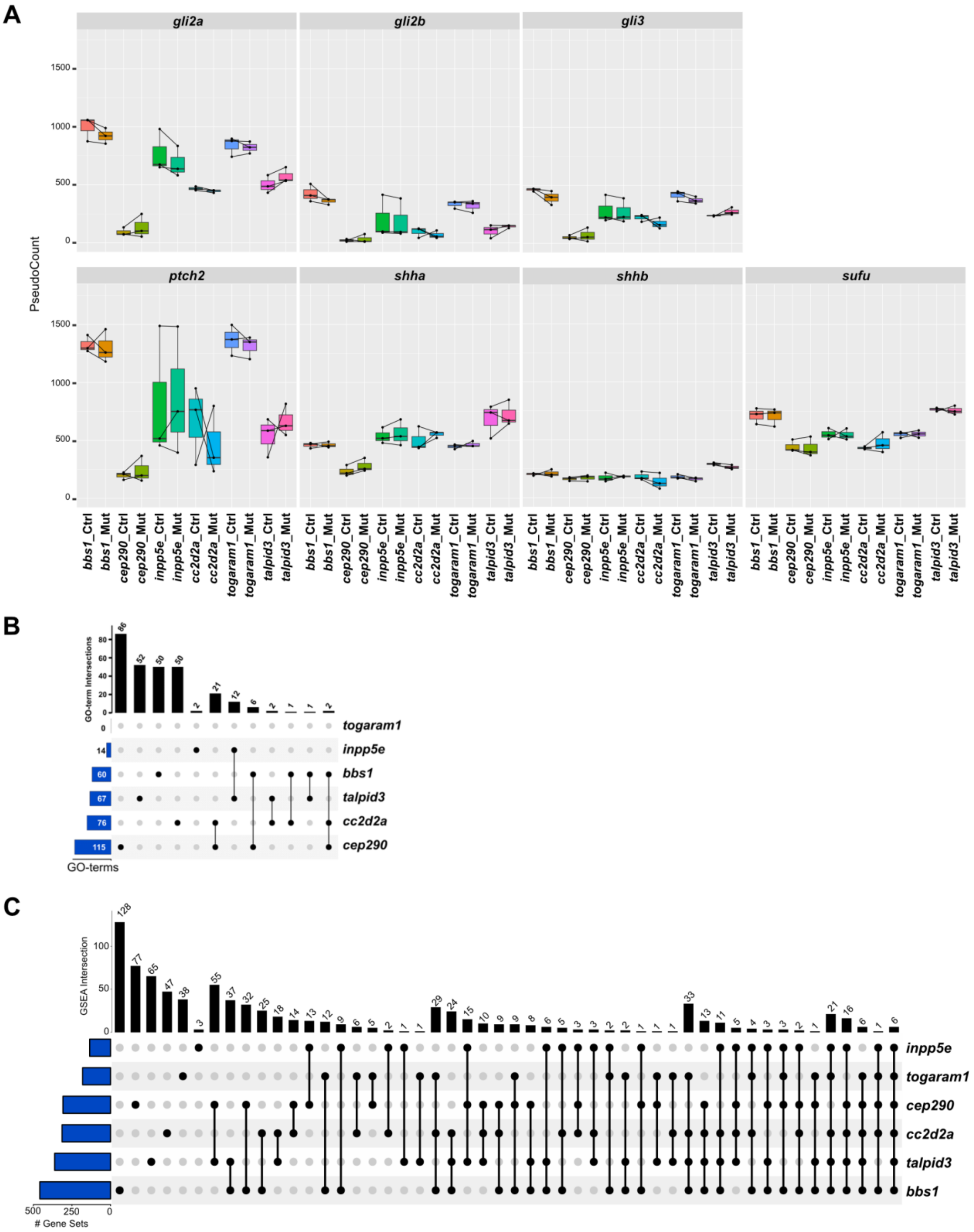

Biology Open • Supplementary information

Fig. S3. Comparison of transcriptomic changes in multiple zebrafish ciliopathy mutants

(A) Box plots showing the normalised expression levels of key actors of the Shh pathway in the different mutant lines and their respective controls in the whole larval tissue analysis. Each data point represents an individual sample (of pooled larvae, see details in methods), where paired mutant/control sibling samples are connected by a black line. Box plots represent Q1-Q3 with median (thick line). **(B)** The upset plot visually represents Gene Ontology (GO) terms significantly overrepresented in the RNAseq analysis (adj. P-value < 0.05). The blue bar on the left denotes the total number of identified terms, while the black bars on top indicate the count of intersecting terms (dots below indicating the respective mutants). **(C)** Gene set enrichment analysis highlighting substantial commonality between the mutant lines. The blue bars on the left indicate the number of gene ontology terms per mutant/control pair while the black bar on top indicates the number of shared terms found in several samples indicated by the black dots. Detail on the ontology terms in each intercept can be found in Table S5.

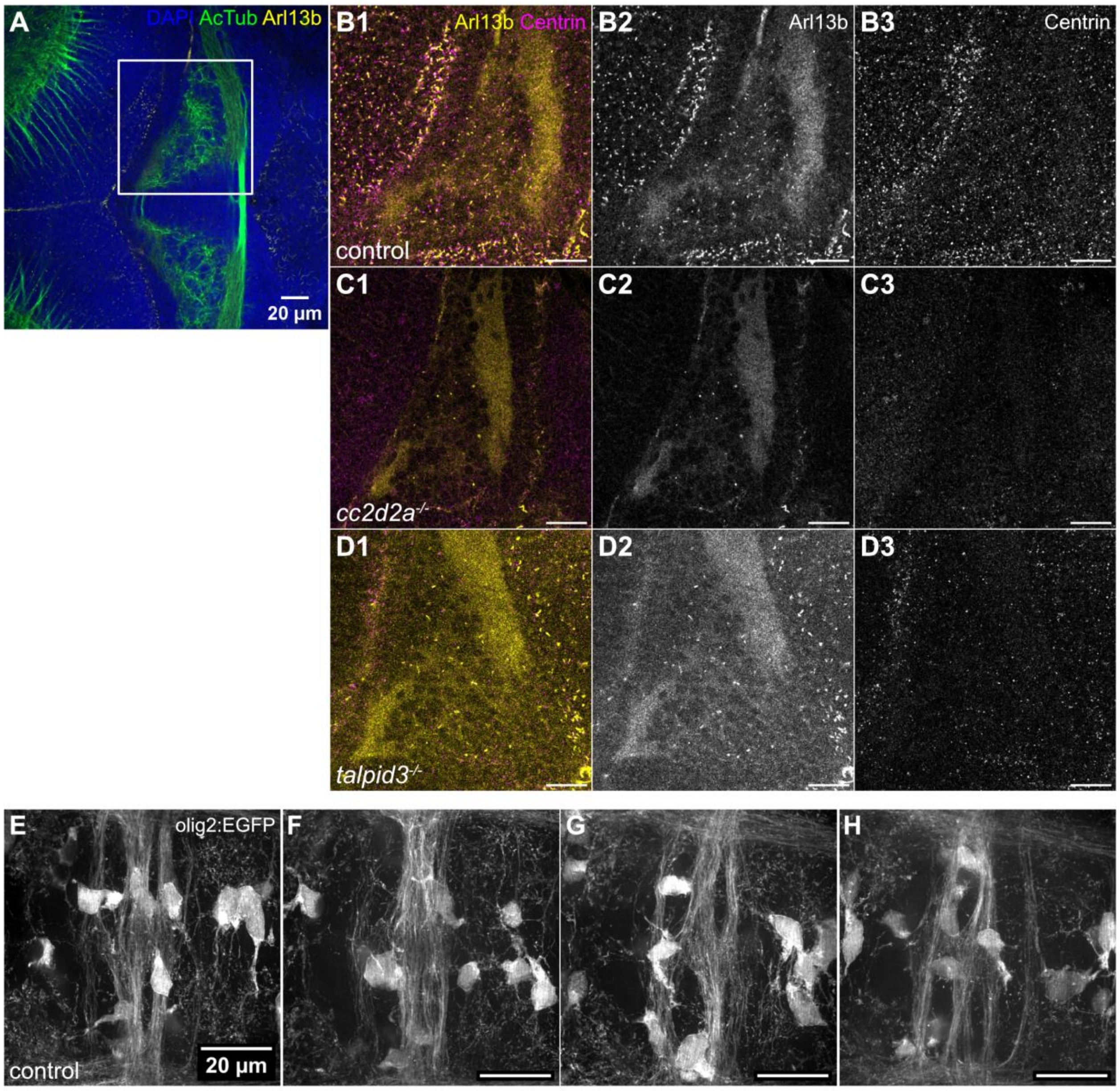

Fig. S4. Confirmation of ciliary defects in *cc2d2a* and *talpid3* mutant cerebella using antibodies against endogenous Arl13b and variability of eurydendroid axons in wildtype larvae

(A-D) Whole-mount single optical slice confocal immunofluorescence images showing Arl13b-positive primary cilia labelled with anti-Arl13b (Arl13b – yellow) and basal bodies labelled with anti-Centrin (Centrin – magenta) within the cerebellum of control (B, n = 13, N = 2), *cc2d2a*$^{-/-}$ (C, n = 6, N = 2) and *talpid3*$^{-/-}$ (D, n = 6, N = 2) larvae at 5 dpf. Boxed region in (A) serves for orientation showing location of images in (B-D). Note a reduction in Arl13b-positive primary cilia in mutants compared to controls in both mutants. **(E-H)** Representative whole-mount maximum projection confocal images showing variable morphologies of EGFP-positive eurydendroid cell axons in wildtype (WT) *Tg(olig2:EGFP)* larvae at 5 dpf. All images show a dorsal view of 5 dpf larvae with anterior to the left. All scale bars are 20 µm. N denotes the number of independent experiments (i.e. larvae from independent clutches) used for analysis.

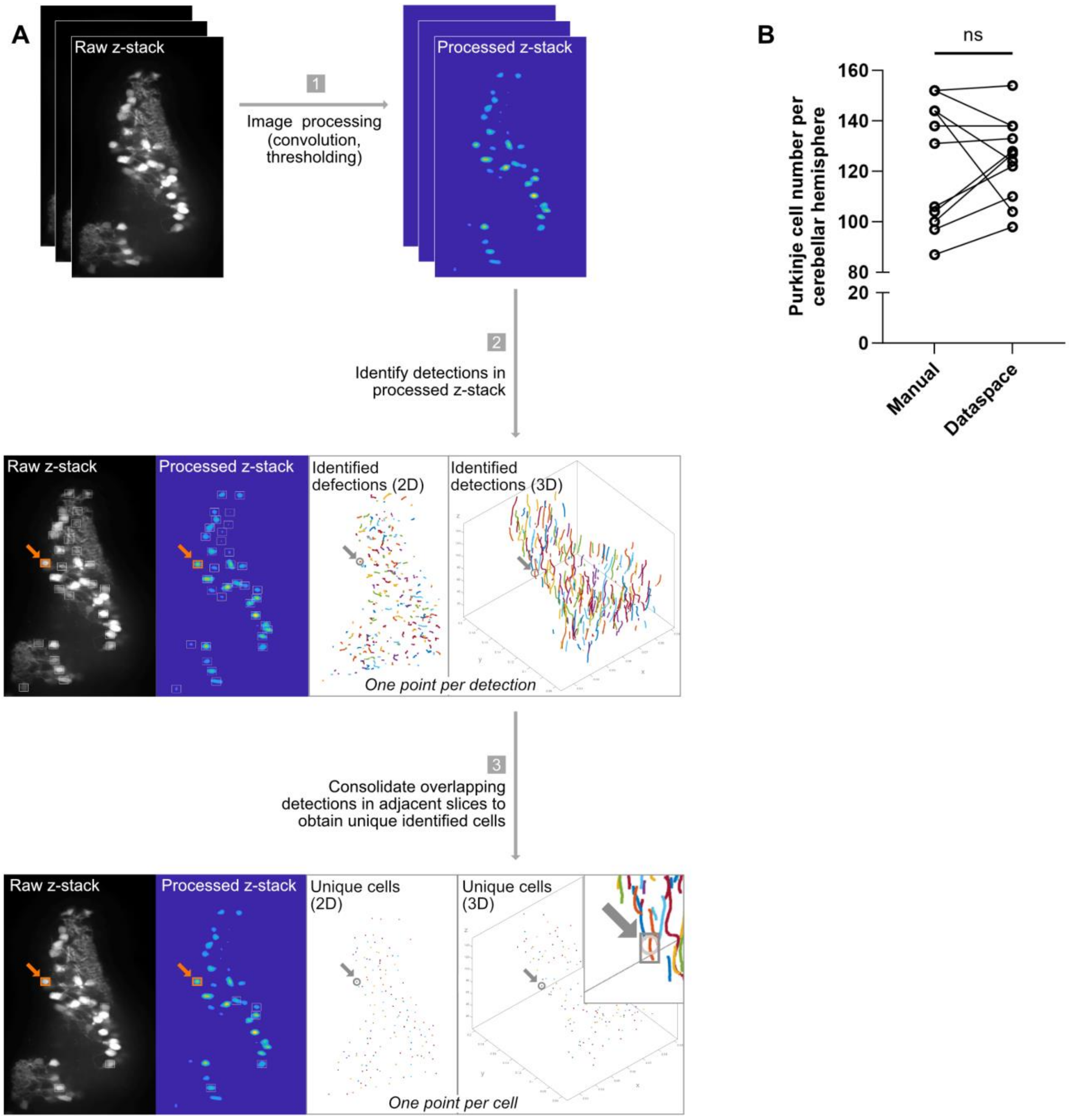

Fig. S5. Cell number quantification using Dataspace

(A) Schematic showing analysis pipeline for cell number quantification using Dataspace. **(B)** Scatter plot showing comparison between the number of Purkinje cells in one cerebellar hemisphere quantified manually or using Dataspace. Each paired data point represents manual and Dataspace quantification of the same larva. ns, not significant. Paired t test. n = 11 larvae, P = 0.76.

Biology Open • Supplementary information

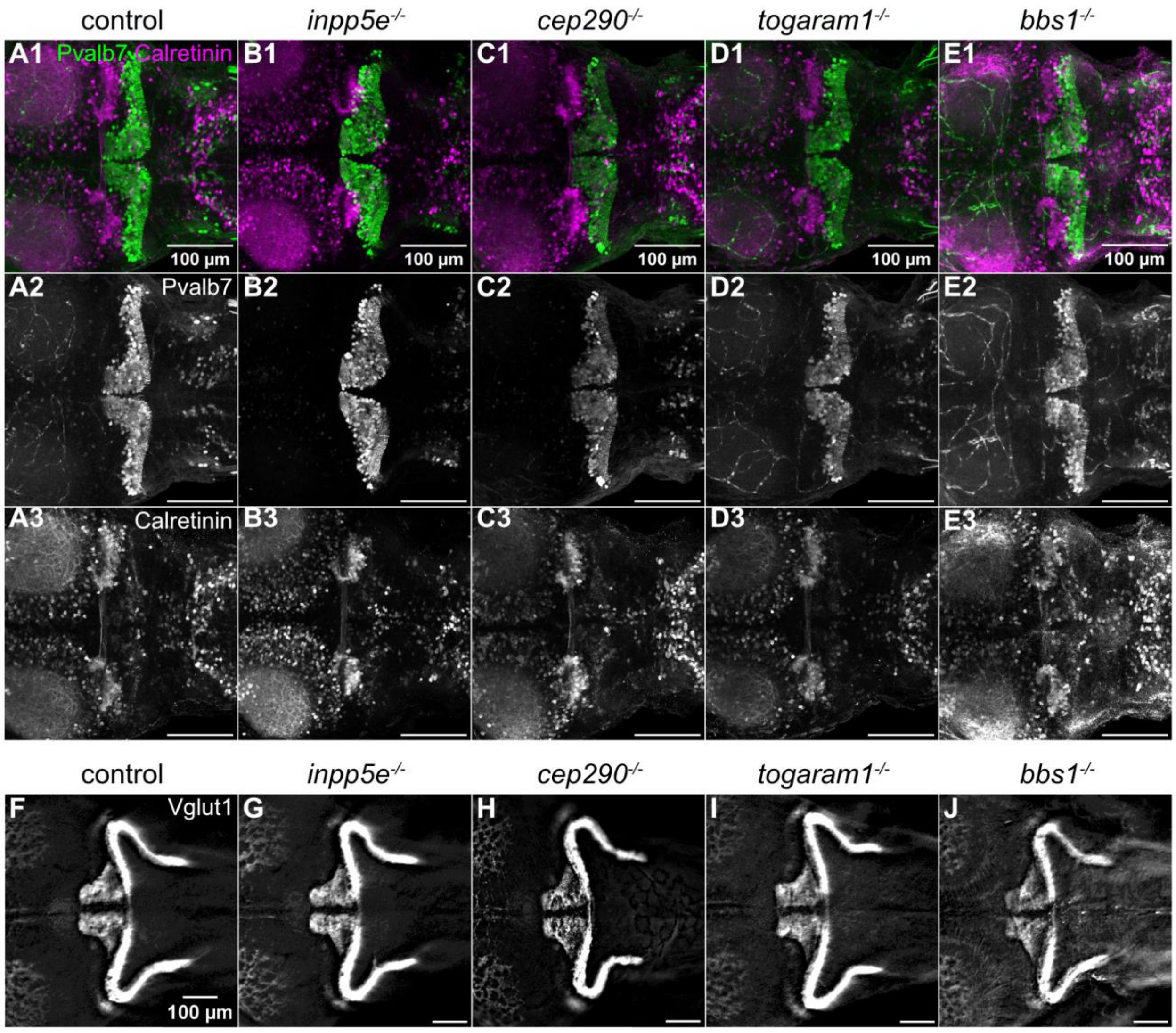

Fig. S6. Cerebellar development is unaffected in JBTS mutants

(A-E) Whole-mount maximum projection confocal immunofluorescence images showing Pvalb7-positive Purkinje cells (Pvalb7 – green) and Calretinin-positive eurydendroid cells (Calretinin – magenta) in zygotic *inpp5e*$^{-/-}$ (n = 5), *cep290*$^{-/-}$ (n = 6), *togaram1*$^{-/-}$ (n = 9) and *bbs1*$^{-/-}$ (n = 7) larvae compared to controls (n = 24) at 5 dpf. The morphology of the Purkinje and eurydendroid cell layers in mutants is comparable to controls. **(F-J)** Whole-mount single optical slice widefield immunofluorescence images showing parallel fibres labelled with anti-Vglut1 (Vglut1 – grey) in zygotic *inpp5e*$^{-/-}$ (n = 15), *cep290*$^{-/-}$ (n = 12), *togaram1*$^{-/-}$ (n = 7) and *bbs1*$^{-/-}$ (n = 10) larvae compared to controls (n = 42) at 5 dpf. The morphology of parallel fibres is unaffected in mutants compared to controls. All images show a dorsal view of 5 dpf larvae with anterior to the left. Scale bars are 100 µm. Mutant images are representative of N = 1 and control images are representative of N = 4 independent experiments (i.e. larvae from independent clutches).

Biology Open • Supplementary information

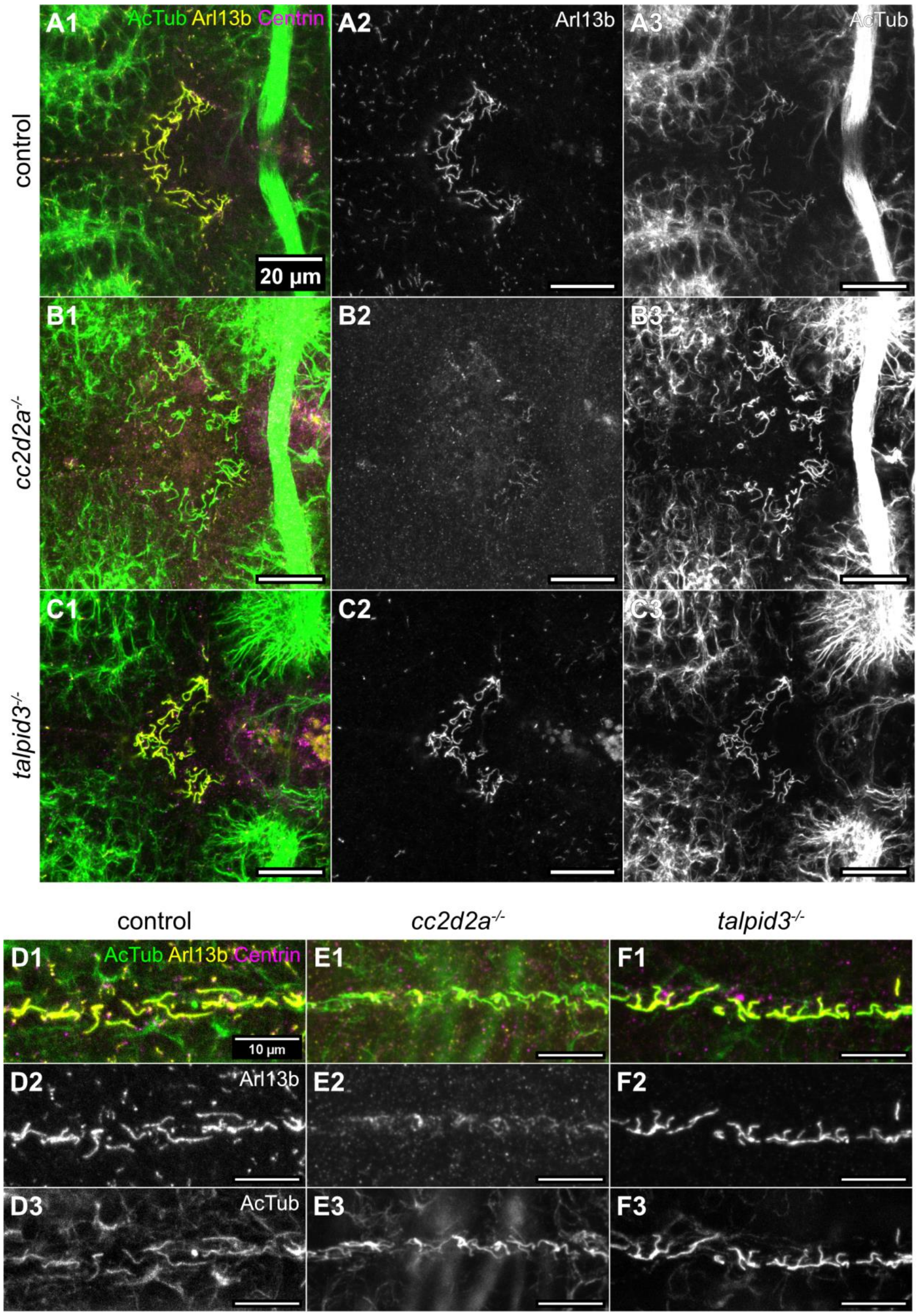

Biology Open • Supplementary information

Fig. S7. Abnormal motile cilia in the brain of *cc2d2a* and *talpid3* mutants

(A-F) Whole-mount maximum projection confocal immunofluorescence images of motile cilia in the forebrain ventricle (A-C) and midbrain ventricle (D-F) of control (n = 13 in A, n = 14 in D, N = 2), mz *cc2d2a*$^{-/-}$ (n = 9 in B, n = 8 in E, N = 1) and zygotic *talpid3*$^{-/-}$ (n = 6 in C, n = 5 in F, N = 2) larvae at 5 dpf, labelled with anti-acetylated tubulin (AcTub – green) and anti-Arl13b (Arl13b – yellow). Anti-Centrin was used to label basal bodies (Centrin – magenta). Motile cilia in *cc2d2a*$^{-/-}$ larvae show reduced immunostaining for Arl13b but appear unaffected in *talpid3*$^{-/-}$ larvae. All images show a dorsal view of 5 dpf larvae with anterior to the left. Scale bars are 20 µm in (A-C) and 10 µm in (D-F). N denotes the number of independent experiments (i.e. larvae from independent clutches) used for analysis.

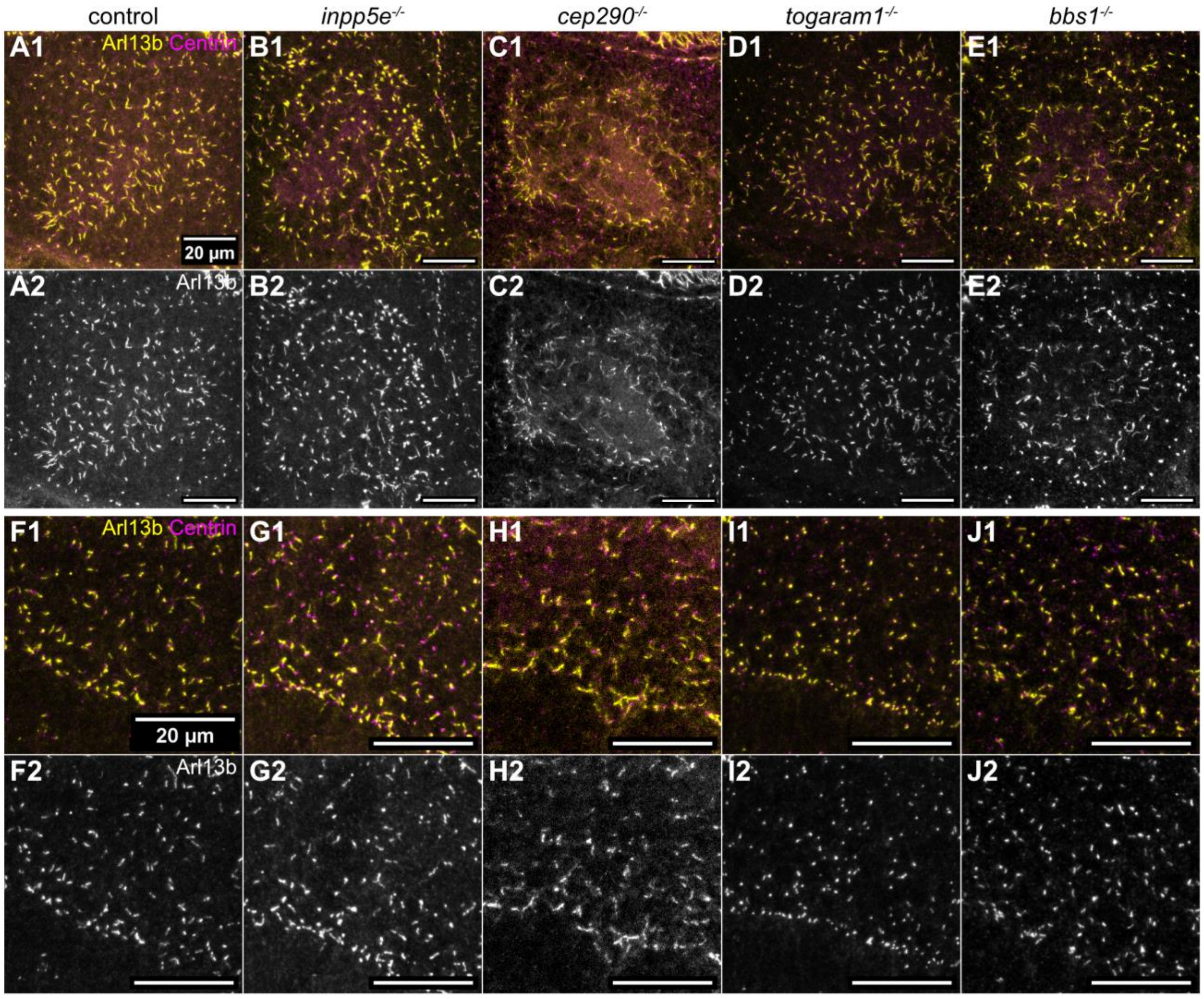

Fig. S8. Abnormal primary cilia in the brain of JBTS mutants

(A-J) Whole-mount maximum projection confocal immunofluorescence images of primary cilia in the forebrain parenchyma (A-E) and midbrain parenchyma (F-J) of zygotic *inpp5e*$^{-/-}$ (n = 11), *cep290*$^{-/-}$ (n = 5), *togaram1*$^{-/-}$ (n = 10) and *bbs1*$^{-/-}$ (n = 13) larvae compared to controls (n = 20) at 5 dpf, labelled using anti-Arl13b (Arl13b – yellow) with anti-Centrin to label basal bodies (Centrin – magenta). Primary cilia are present in all mutants, with variable reductions in length or number. All images show a dorsal view of 5 dpf larvae with anterior to the left. Scale bars are 20 µm. *inpp5e*$^{-/-}$ and *bbs1*$^{-/-}$ mutant images are representative of N = 2, *cep290*$^{-/-}$ and *togaram1*$^{-/-}$ are representative of N = 1 and control images are representative of N = 5 independent experiments (i.e. larvae from independent clutches).

Biology Open • Supplementary information

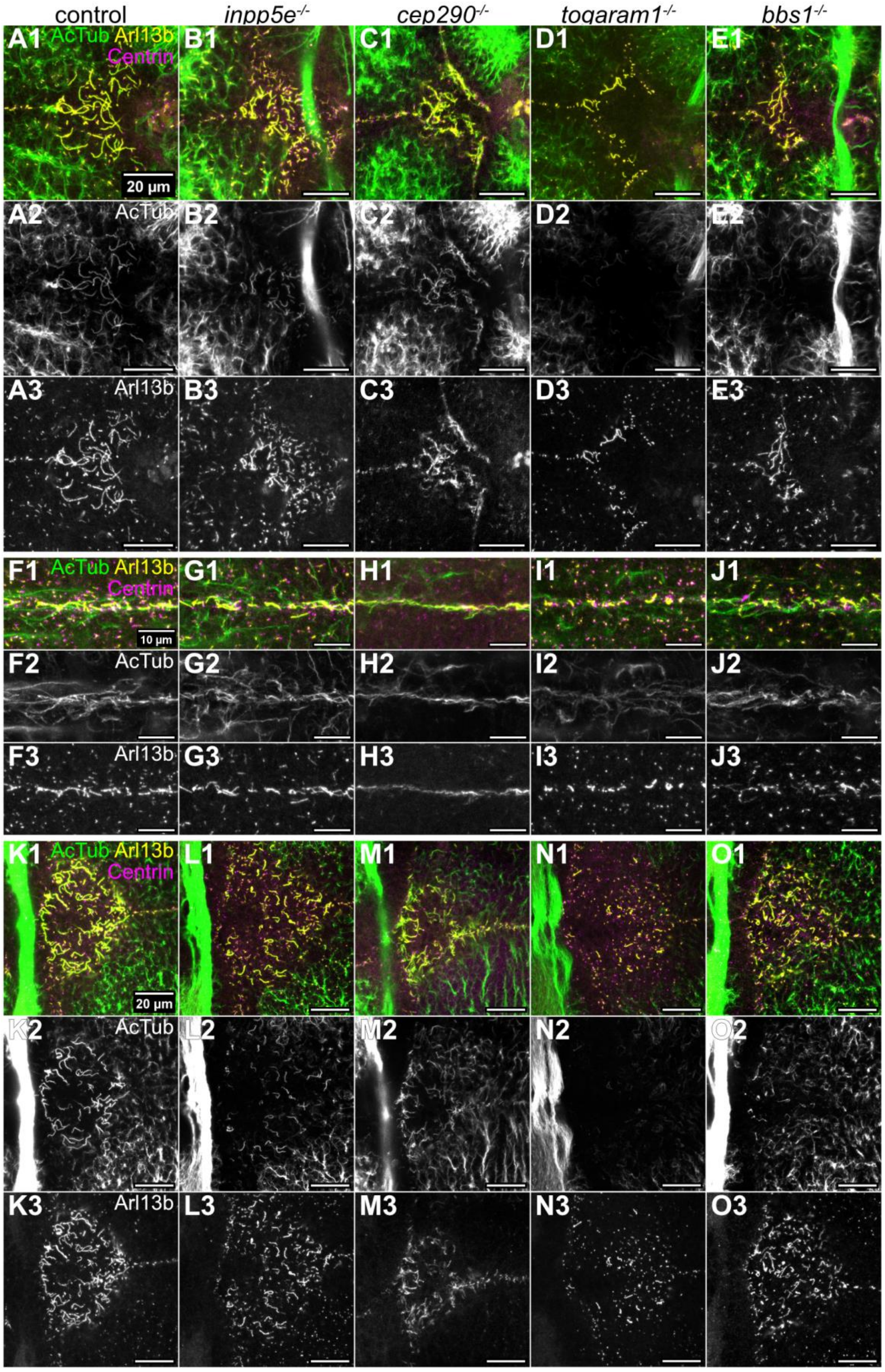

Biology Open • Supplementary information

Fig. S9. Abnormal motile cilia in the brain of JBTS mutants

(A-O) Whole-mount maximum projection confocal immunofluorescence images of motile cilia in the forebrain ventricle (A-E), midbrain ventricle (F-J) and hindbrain ventricle (K-O) of zygotic *inpp5e*$^{-/-}$ (n = 6), *cep290*$^{-/-}$ (n = 5), *togaram1*$^{-/-}$ (n = 10) and *bbs1*$^{-/-}$ (n = 12) larvae compared to controls (n = 18) at 5 dpf. Motile cilia are labelled with anti-acetylated tubulin (AcTub – green) and anti-Arl13b (Arl13b – yellow). Basal bodies are labelled with anti-Centrin (Centrin – magenta). Motile cilia are present in all mutants and show variable mild phenotypes, with *togaram1*$^{-/-}$ being the most severely affected. All images show a dorsal view of 5 dpf larvae with anterior to the left. Scale bars are 20 µm in (A-E) and (K-O) and 10 µm in (F-J). *inpp5e*$^{-/-}$ and *bbs1*$^{-/-}$ mutant images are representative of N = 2, *cep290*$^{-/-}$ and *togaram1*$^{-/-}$ are representative of N = 1 and control images are representative of N = 5 independent experiments (i.e. larvae from independent clutches).

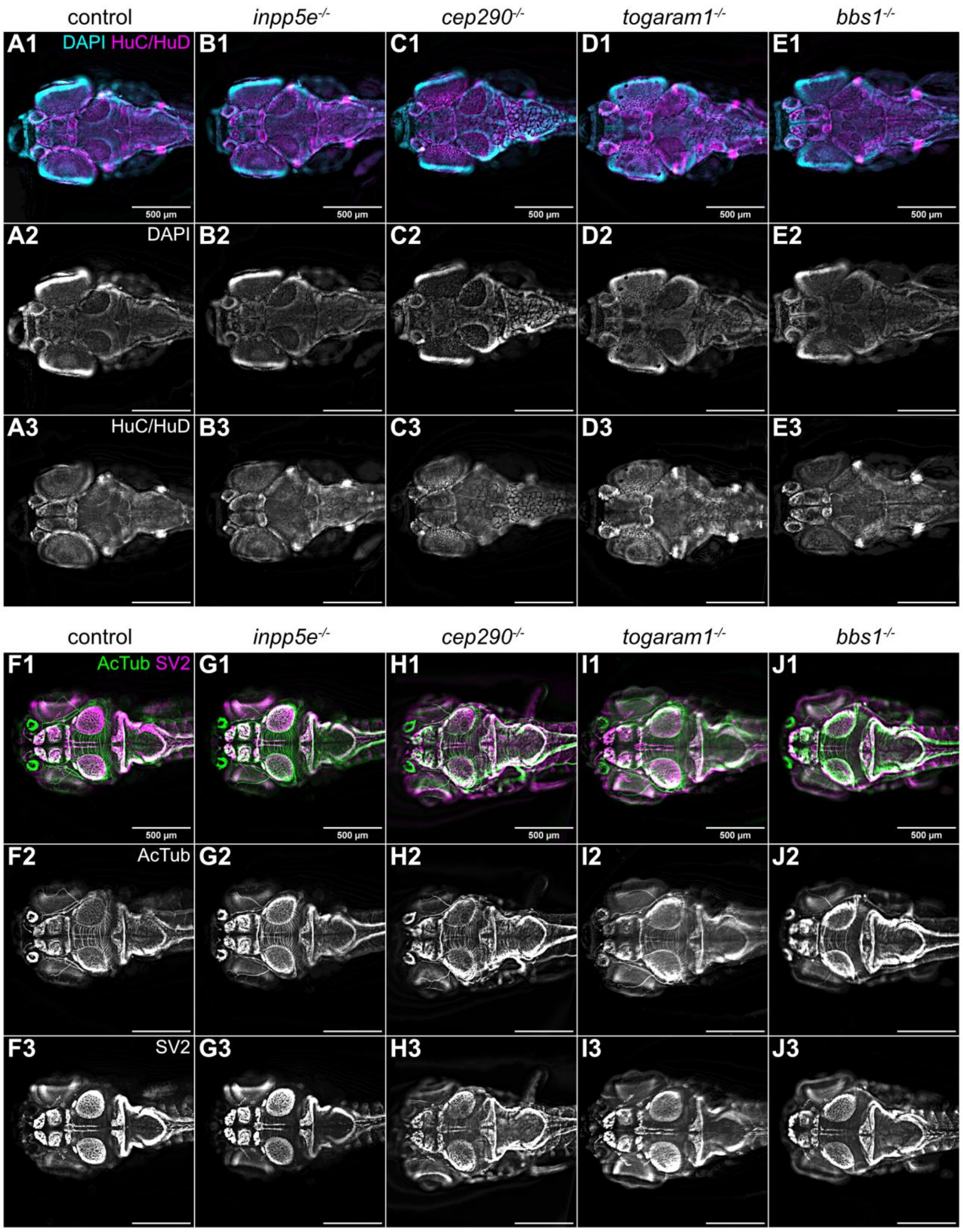

Fig. S10. CNS development is unaffected in JBTS mutants

(A-E) Whole-mount single optical slice widefield immunofluorescence images showing brain morphology by immunostaining with anti-HuC/HuD (HuC/HuD – magenta) and nuclei counterstaining with DAPI (DAPI – cyan) in zygotic *inpp5e*$^{-/-}$ (n = 10), *cep290*$^{-/-}$ (n = 9), *togaram1*$^{-/-}$ (n = 6) and *bbs1*$^{-/-}$ (n = 11) larvae compared to controls (n = 30) at 5 dpf. The morphology of the brain in mutants is comparable to controls. **(F-J)** Whole-mount single optical slice widefield immunofluorescence images showing axonal tracts labelled with anti-acetylated tubulin (AcTub – green) and synaptic neuropil labelled with anti-SV2 (SV2 – magenta) in zygotic *inpp5e*$^{-/-}$ (n = 10), *cep290*$^{-/-}$ (n = 6), *togaram1*$^{-/-}$ (n = 6) and *bbs1*$^{-/-}$ (n = 8) larvae compared to controls (n = 33) at 5 dpf. The organisation of axon tracts and synaptic neuropil is unaffected in mutants compared to controls. All images show a dorsal view of 5 dpf larvae with anterior to the left. Scale bars are 500 µm. Mutant images are representative of N = 1 and control images are representative of N = 4 independent experiments (i.e. larvae from independent clutches).

Biology Open • Supplementary information

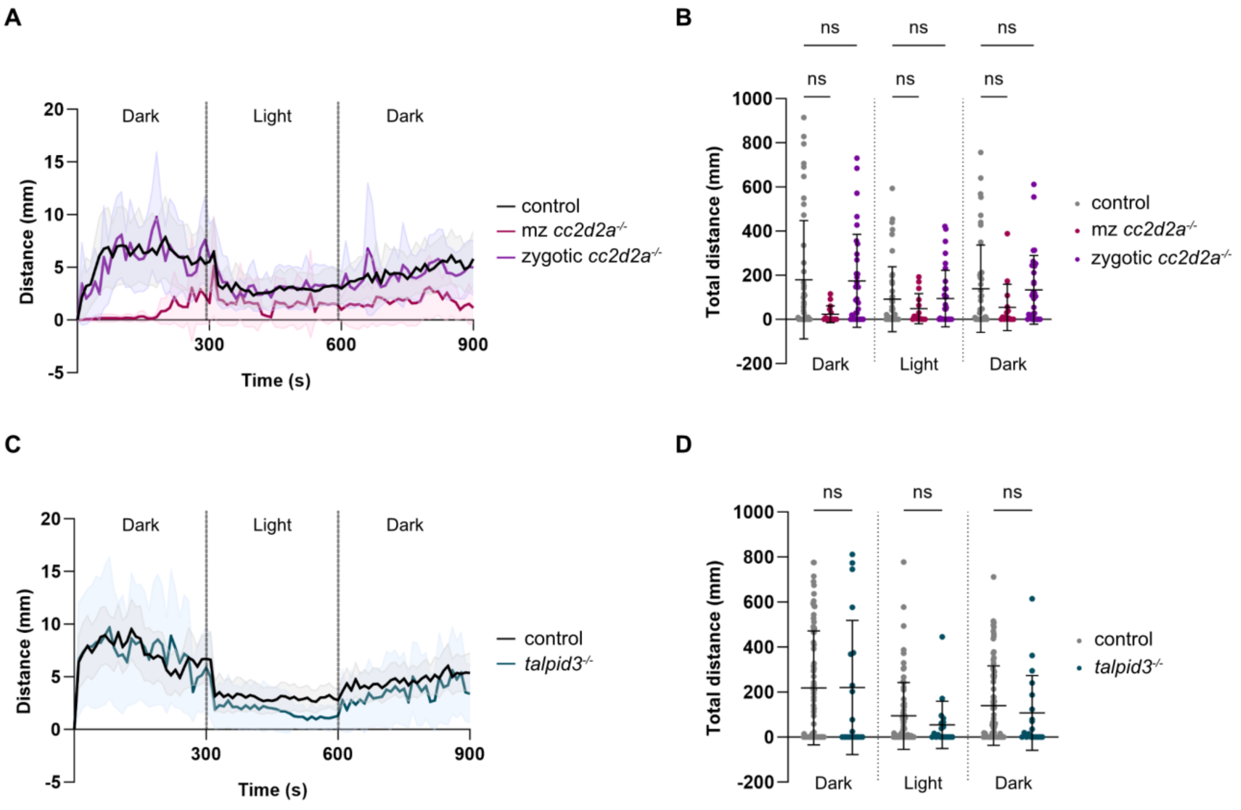

Fig. S11. Swimming behaviour of *cc2d2a* and *talpid3* mutants at 3 dpf

(A) Plot showing mean distance travelled over time during 3 sequential 5-minute periods of darkness, light, darkness in mz *cc2d2a*$^{-/-}$ and zygotic *cc2d2a*$^{-/-}$ larvae compared to controls at 3 dpf. Total distance moved is recorded during 10 second intervals and this distance is plotted over time. Note that while mz *cc2d2a*$^{-/-}$ larvae travel less than controls during both periods of darkness, this difference does not reach statistical significance. Error shown with the coloured areas represent the 95% CI. **(B)** Scatter plot showing the total distance travelled during the 5-minute periods of darkness, light and darkness in mz *cc2d2a*$^{-/-}$ and zygotic *cc2d2a*$^{-/-}$ larvae compared to controls at 3 dpf. Total distance travelled is not significantly reduced but a trend towards decreased distance is visible in mz *cc2d2a*$^{-/-}$ larvae. Error bars are mean±s.d. ns, not significant. Kruskal-Wallis test with post-hoc Dunn's multiple comparisons test. Control n = 47 (N = 3), mz *cc2d2a*$^{-/-}$ n = 15 (N = 1), zygotic *cc2d2a*$^{-/-}$ n = 32 (N = 2) larvae. **(C)** Similar plot as in (A) but for zygotic *talpid3*$^{-/-}$ larvae compared to controls at 3 dpf. The distance travelled throughout the experiment is consistent between mutants and controls. Error shown with the coloured areas represent the 95% CI. **(D)** Scatter plot showing the total distance travelled during the 5-minute periods of darkness, light, darkness in zygotic *talpid3*$^{-/-}$ larvae compared to controls at 3 dpf. Total distance is not significantly reduced in zygotic *talpid3*$^{-/-}$ larvae compared to controls. Error bars are mean±s.d. ns, not significant. Mann-Whitney test. Control n = 77 larvae (N = 3), *talpid3*$^{-/-}$ n = 19 larvae (N = 3). N denotes the number of independent experiments (i.e. larvae from independent clutches) used for analysis.

Biology Open • Supplementary information

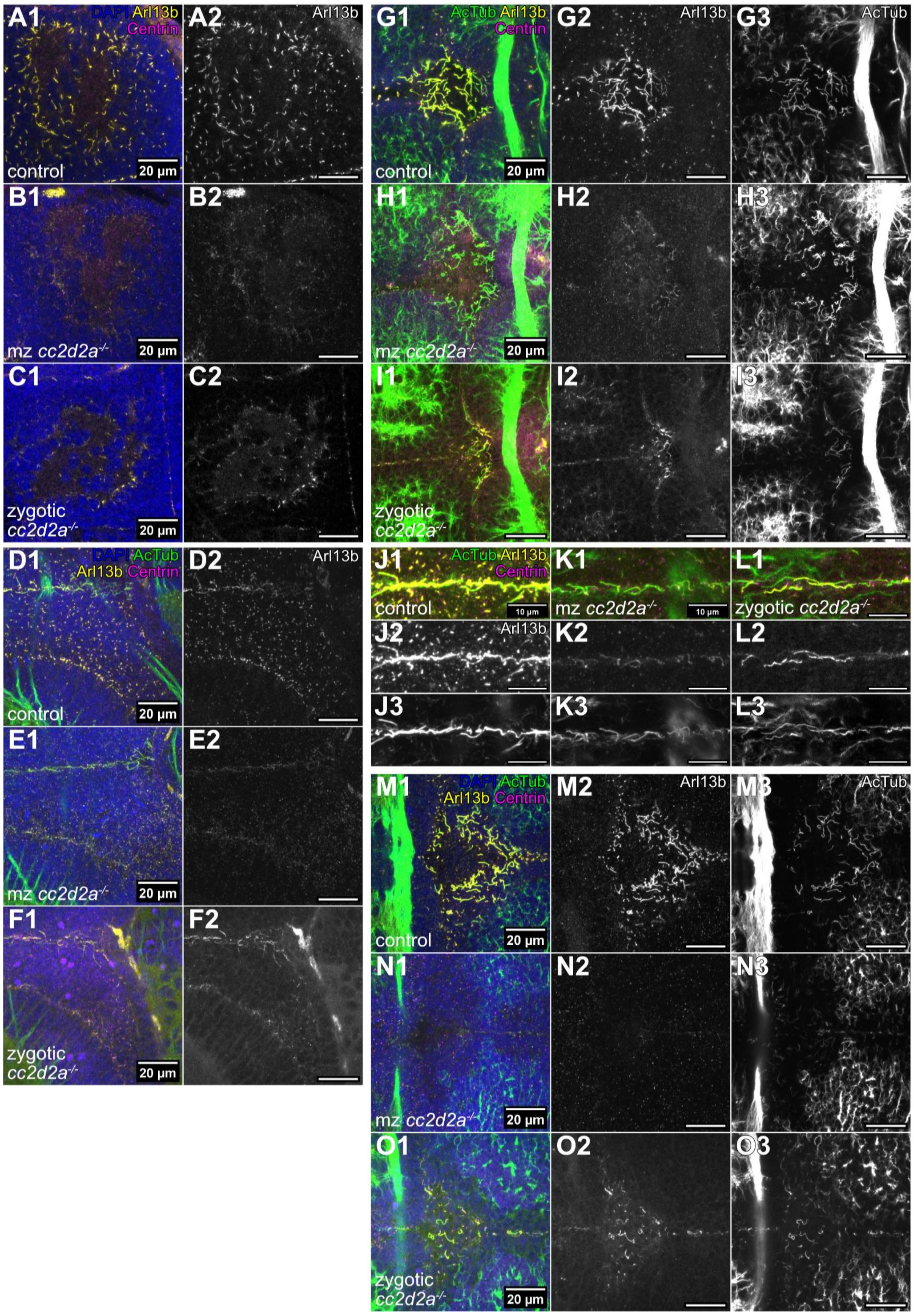

Fig. S12. Comparing the ciliary phenotype in the brain of mz *cc2d2a* and zygotic *cc2d2a* mutants at 5 dpf

(A-O) Whole-mount maximum projection confocal immunofluorescence images of primary cilia in the forebrain parenchyma (A, n = 8; B, n = 9; C, n = 4) and midbrain parenchyma (D, n = 7; E, n = 9; F, n = 4), and of motile cilia in the forebrain ventricle (G, n = 8; H, n = 9; I, n = 4), midbrain ventricle (J, n = 9; K, n = 8; L, n = 4) and hindbrain ventricle (M, n = 6; N, n = 8; O, n = 4) of control, mz *cc2d2a*$^{-/-}$ and zygotic *cc2d2a*$^{-/-}$ larvae at 5 dpf. Primary cilia are labelled with anti-Arl13b (Arl13b – yellow) while motile cilia are labelled with anti-acetylated tubulin (AcTub – green) and anti-Arl13b (Arl13b – yellow). Basal bodies are labelled with anti-Centrin (Centrin – magenta). Compared to controls, zygotic *cc2d2a*$^{-/-}$ have fewer primary cilia in all brain regions, and this reduction is less pronounced than in mz *cc2d2a*$^{-/-}$. All images show a dorsal view of 5 dpf larvae with anterior to the left. Scale bars are 20 µm in (A-I) and (M-O) and 10 µm in (J-L). Images are representative of N = 1 independent experiment (i.e. larvae from independent clutches).

Biology Open • Supplementary information

Table S1. Overview of analysed JBTS and BBS zebrafish mutants

Table giving details on the genetic background, method used to generate mutant, mutation, reported ciliopathy-related phenotypes and references for each mutant. IP = incompletely penetrant phenotype.

Mutant	Genetic Background	Generation	Mutation	Gross phenotypes	References
cc2d2a^{w38}	Mixed (AB, unknown)	ENU mutagenesis	Chr 23: 2216374 G>A (GRCz11) p.Trp628*	body curvature, renal cysts (IP) and retinal degeneration	Owens et al. 2008; Gorden et al. 2008; Bachmann-Gagescu et al. 2011; Ojeda Naharros et al. 2017
cep290^{fh297}	Mixed (AB, Tü)	ENU mutagenesis	Chr 25: 17956127 C>T (GRCz11) p.Gln1217*	variable body curvature (IP) and mild and variable retinal degeneration	Lessieur et al. 2019, Fig. S2
inpp5e^{zh506}	Mixed (WIK, Tü)	CRISPR gene editing	Chr 5: 65081517 – 65081525 del, 65081585 – 65081613 del (GRCz11) p.Ser165*	body curvature and renal cysts	Fig. S1
talpid3^{i264}	Mixed (AB, unknown)	ZFN-mediated targeted mutagenesis	Chr 17: 11254088 – 11254089 ins CCTGCTTCCTCAAGCCC (GRCz11) p.Phe555GlyFs*14	body curvature*, renal cysts	Ben et al. 2011; Ojeda Naharros et al. 2018
togaram1^{zh510}	Tü	CRISPR gene editing	Chr 17: 645504 - 645920 del ins TGTGAGGAAA (GRCz11) p.Pro1130ProFs*72	body curvature, renal cysts	Latour et al. 2020
bbs1^{k742}	Mixed (AB, Tü)	CRISPR gene editing	Chr 21: 27467605 – 27467609 ins GCCAA (GRCz11) p.Ala137GlyFs*5	variable body curvature (IP), retinal degeneration	Masek et al. 2022

*mz mutants only

Biology Open • Supplementary information

Table S2. Overview differential expression analysis data

The table provides a comprehensive overview of the results of the DESeq2 differential expression analysis for each mutant compared to its respective control. Each workbook in the table corresponds to a single mutant and includes detailed information such as test statistics, gene names and pseudo counts.

Available for download at
https://journals.biologists.com/bio/article-lookup/doi/10.1242/bio.060421#supplementary-data

Table S3. Intercept of individual differentially expressed genes across different lines

This list provides an overview of the intersection at the individual gene level, emphasizing genes that exhibit significant differential expression (adj. P-value < 0.05) across various mutants.

Available for download at
https://journals.biologists.com/bio/article-lookup/doi/10.1242/bio.060421#supplementary-data

Table S4. Results overrepresentation analysis including intercept across all tested lines

The first six workbooks present enriched GO terms (adj. P-value < 0.05) specific to individual mutants, identified by overrepresentation analysis. Subsequent lists highlight the overlap of terms between different mutants. A GO semantic similarity measure, facilitated by the GOSemSim R package (Yu et al. 2010), was used to assign values close to 1 for highly semantic terms and values close to zero for non-semantic terms.

Available for download at
https://journals.biologists.com/bio/article-lookup/doi/10.1242/bio.060421#supplementary-data

Biology Open • Supplementary information

Table S5. Results gene set enrichment analysis including intercept across all tested lines

The first six workbooks present enriched GO terms (adj. P-value < 0.05) specific to individual mutants, identified by gene set enrichment analysis. Subsequent lists highlight the overlap of terms between different mutants. A GO semantic similarity measure, facilitated by the GOSemSim R package (Yu et al. 2010), was used to assign values close to 1 for highly semantic terms and values close to zero for non-semantic terms.

Available for download at
https://journals.biologists.com/bio/article-lookup/doi/10.1242/bio.060421#supplementary-data

Table S6. Quantification of Arl13b+ cilia in the Purkinje and eurydendroid cell layers in control vs. *cc2d2a* vs. *talpid3* mutants at 5 dpf with statistics

Mean number and s.d. of Arl13b+ cilia in the Purkinje cell layer and *olig2*+ eurydendroid cell layer in control, *cc2d2a*$^{-/-}$ and *talpid3*$^{-/-}$ at 5 dpf. P-values were calculated using Welch's ANOVA with post-hoc Dunnett's T3 multiple comparisons test.

	**Purkinje cell layer**			**Eurydendroid cell layer (*olig2+*)**		
	**control**	***cc2d2a*$^{-/-}$**	***talpid3*$^{-/-}$**	**control**	***cc2d2a*$^{-/-}$**	***talpid3*$^{-/-}$**
**mean**	63.92	8.50	25.47	75.16	10.40	25.69
**s.d.**	17.07	7.23	10.91	35.28	5.51	7.22
**P**		<0.0001	<0.0001		<0.0001	<0.0001

Table S7. Quantification of different brain areas in control vs. *cc2d2a* mutants at 5 dpf with statistics

Mean normalised area and s.d. (in µm) of the forebrain (FB), midbrain (MB) and hindbrain (HB) of control and *cc2d2a*$^{-/-}$ at 5 dpf. Unpaired t test.

	**control**			***cc2d2a*$^{-/-}$**		
	FB	MB	HB	FB	MB	HB
**mean**	0.1719	0.4103	0.4157	0.1693	0.4029	0.424
**s.d.**	0.0082	0.0242	0.0280	0.0091	0.0242	0.0294
**P**				0.370	0.378	0.400

Table S8. Quantification of different brain areas in control vs. *talpid3* mutants at 5 dpf with statistics

Mean normalised area and s.d. (in µm) of the forebrain (FB), midbrain (MB) and hindbrain (HB) of control and *talpid3*$^{-/-}$ at 5 dpf. Unpaired t test.

	**control**			***talpid3*$^{-/-}$**		
	**FB**	**MB**	**HB**	**FB**	**MB**	**HB**
**mean**	0.1894	0.4386	0.3658	0.1836	0.4383	0.3700
**s.d.**	0.0106	0.0251	0.0224	0.0083	0.0185	0.0184
**P**				0.0456	0.9636	0.5011

Biology Open • Supplementary information

Table S9. Mean total distance travelled in controls, mz *cc2d2a* mutants and zygotic *cc2d2a* mutants at 3 dpf and 6 dpf with statistics

Mean total distance and s.d. (in mm) travelled during 5-minute subsequent periods of darkness (D1), light (L), darkness (D2) in control, mz *cc2d2a*$^{-/-}$ and zygotic *cc2d2a*$^{-/-}$ larvae at 3 dpf and 6 dpf. Kruskal-Wallis test with post-hoc Dunn's multiple comparisons test.

		control			mz *cc2d2a*$^{-/-}$			zygotic *cc2d2a*$^{-/-}$		
		D1	L	D2	D1	L	D2	D1	L	D2
3 dpf	mean	179.7	91.9	138.7	23.7	48.1	54.7	174.7	94.4	133.6
	s.d.	267.7	146.9	197.4	37.8	68.0	104.8	210.5	128.3	155.6
	P				0.1244	>0.9999	0.3585	0.7405	>0.9999	0.8422
6 dpf	mean	571.7	245.4	582.9	134.4	182.6	258.9	227.5	166.5	302.8
	s.d.	175.2	162.7	167.2	169.4	298.8	212.3	205.1	181.5	191.0
	P				<0.0001	0.0331	<0.0001	<0.0001	0.0121	<0.0001

Table S10. Mean total distance travelled in controls and *talpid3* mutants at 3 dpf and 6 dpf with statistics

Mean total distance and s.d. (in mm) travelled during 5-minute subsequent periods of darkness (D1), light (L), darkness (D2) in control and *talpid3*$^{-/-}$ larvae at 3 dpf and 6 dpf. For 3 dpf larvae, P-values were calculated using the Mann-Whitney test and for 6 dpf larvae, P-values were calculated using the unpaired t test.

		control			*talpid3*$^{-/-}$		
		D1	L	D2	D1	L	D2
3 dpf	mean	218.2	94.1	139.9	220.1	54.6	107.4
	s.d.	253.2	148.5	176.7	298.1	105.3	166.4
	P				0.8272	0.3910	0.4312
6 dpf	mean	415.5	242.1	496.6	490.9	273.9	386.7
	s.d.	278.7	239.0	248.1	344.7	256.1	294.6
	P				0.2599	0.5507	0.0615

Table S11. Primary and secondary antibodies used for whole-mount immunofluorescence staining

Primary antibodies

Antibody	Species	Supplier and Catalogue number	Dilution	Fixation
Cep290 C-terminus	Rabbit	Gift from I. Drummond	1:50	80% MeOH/DMSO, 2 hours at RT
Pvalb7	Mouse ascites	Gift from M. Hibi	1:1000	4% PFA, 5 hours at 4°C
γ-Tubulin	Rabbit	GeneTex, GTX113286	1:800	4% PFA, 2 hours at RT
Centrin	Mouse IgG2a	Millipore, clone Z0H5	1:200	4% PFA, 2 hours at RT or 2% TCA, 3 hours at RT
Vglut1	Rabbit	Gift from M. Hibi	1:500	80% MeOH/DMSO, 2 hours at RT
Calretinin	Rabbit	Swant, CR 7697	1:1000	4% PFA, 5 hours at 4°C
Arl13b	Rabbit	Proteintech, 17711-1-AP	1:200	2% TCA, 3 hours at RT
Acetylated tubulin	Mouse IgG2b	Sigma-Aldrich, T7451, clone 611B-1	1:400	2% TCA, 3 hours at RT
HuC/HuD	Mouse IgG2b	Invitrogen, A-21271	1:200	4% PFA, 2 hours at RT
SV2	Mouse IgG1	Dev. Studies Hybridoma Bank	1:200	2% TCA, 3 hours at RT

Secondary antibodies

Antibody	Species	Supplier and Catalogue number	Dilution
Alexa goat anti-mouse IgG 488 Plus	Goat	Life technologies, A32723	1:400
Alexa goat anti-mouse IgG2b 488	Goat	Life technologies, A21141	1:400
Alexa goat anti-mouse IgG1 568	Goat	Life technologies, A21124	1:400
Alexa goat anti-mouse IgG 647	Goat	Life technologies, A21235	1:400
Alexa goat anti-mouse IgG2a 647	Goat	Life technologies, A21241	1:400
Alexa goat anti-rabbit IgG 568	Goat	Life technologies, A11036	1:400
Alexa goat anti-rabbit IgG 647	Goat	Life technologies, A21244	1:400

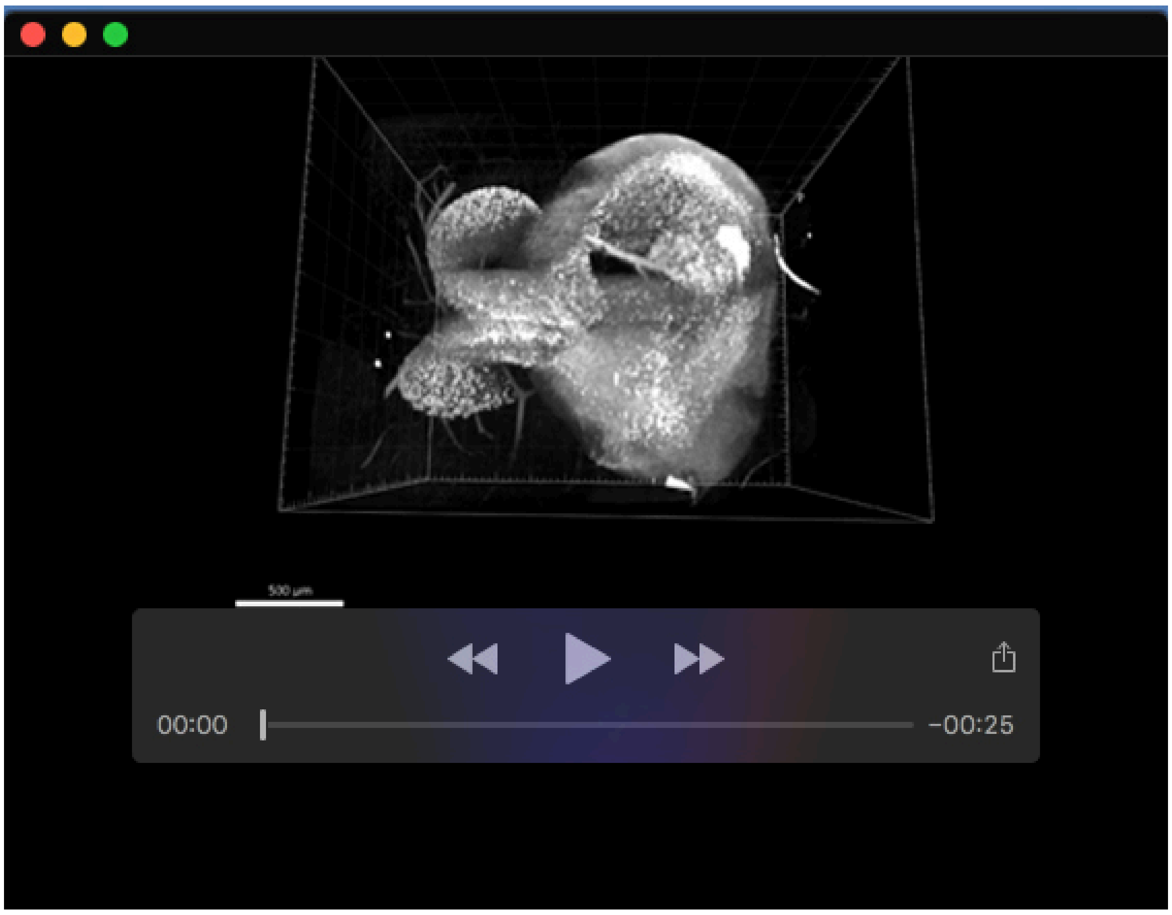

Movie 1. Purkinje cells in control *Tg(tagRFP-T:PC:GCaMP5G)* fish at 11-12 wpf

MesoSPIM fluorescence 3D maximum intensity projection of tagRFP-T-positive Purkinje cells in the cerebellum of *Tg(tagRFP-T:PC:GCaMP5G)* 11-12 wpf control fish, generated using Imaris. The cerebellum is orientated with anterior to the left. Sale bar is 500 µm.

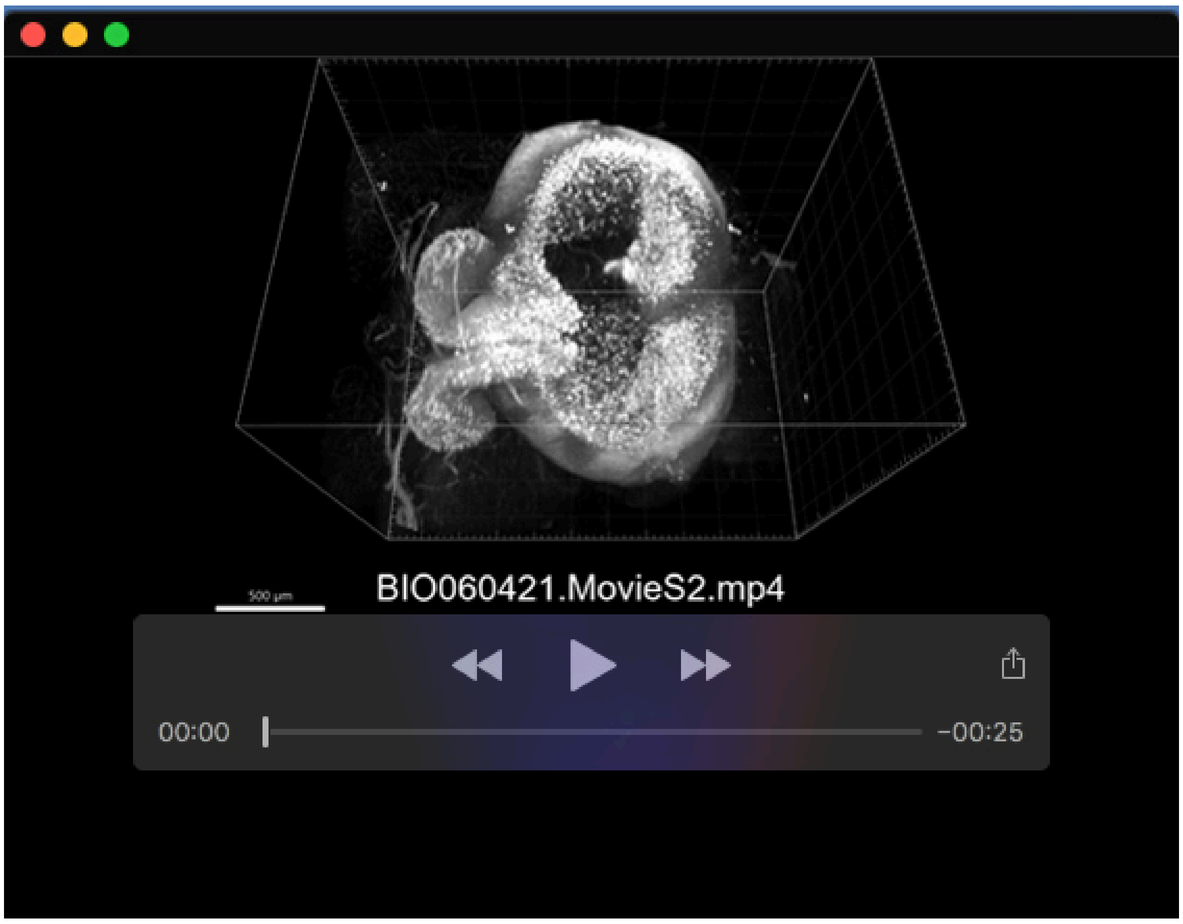

Movie 2. Purkinje cells in *cc2d2a* mutant *Tg(tagRFP-T:PC:GCaMP5G)* fish at 11-12 wpf

MesoSPIM fluorescence 3D maximum intensity projection of tagRFP-T-positive Purkinje cells in the cerebellum of *Tg(tagRFP-T:PC:GCaMP5G)* 11-12 wpf zygotic *cc2d2a* mutant fish, generated using Imaris. The morphology of the Purkinje cell layer is comparable between control and mutant fish. The cerebellum is orientated with anterior to the left. Sale bar is 500 µm.

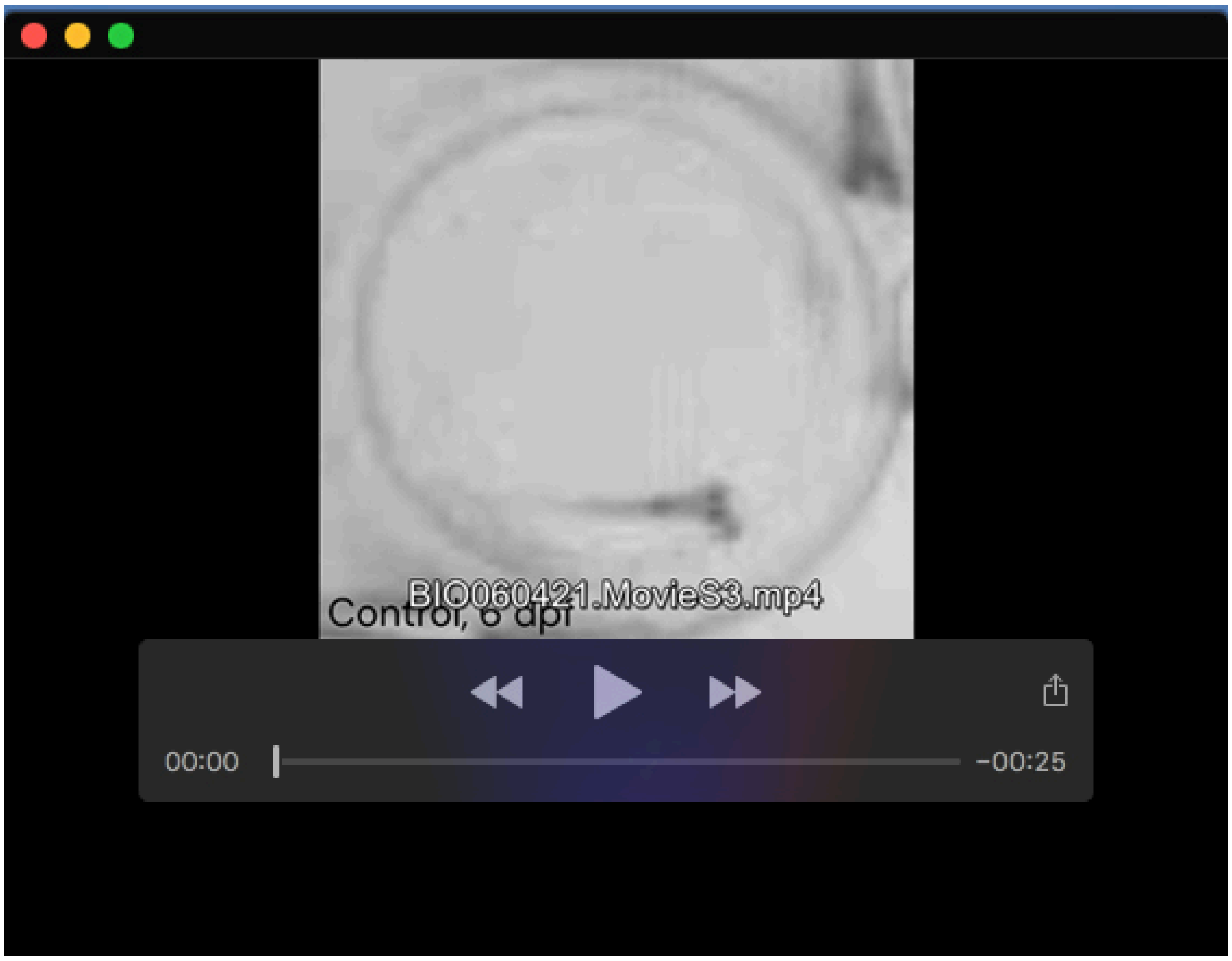

Movie 3. Swimming behaviour of a control larva at 6 dpf

Zebrabox recording of a control larva at 6 dpf, showing normal swimming behaviour.

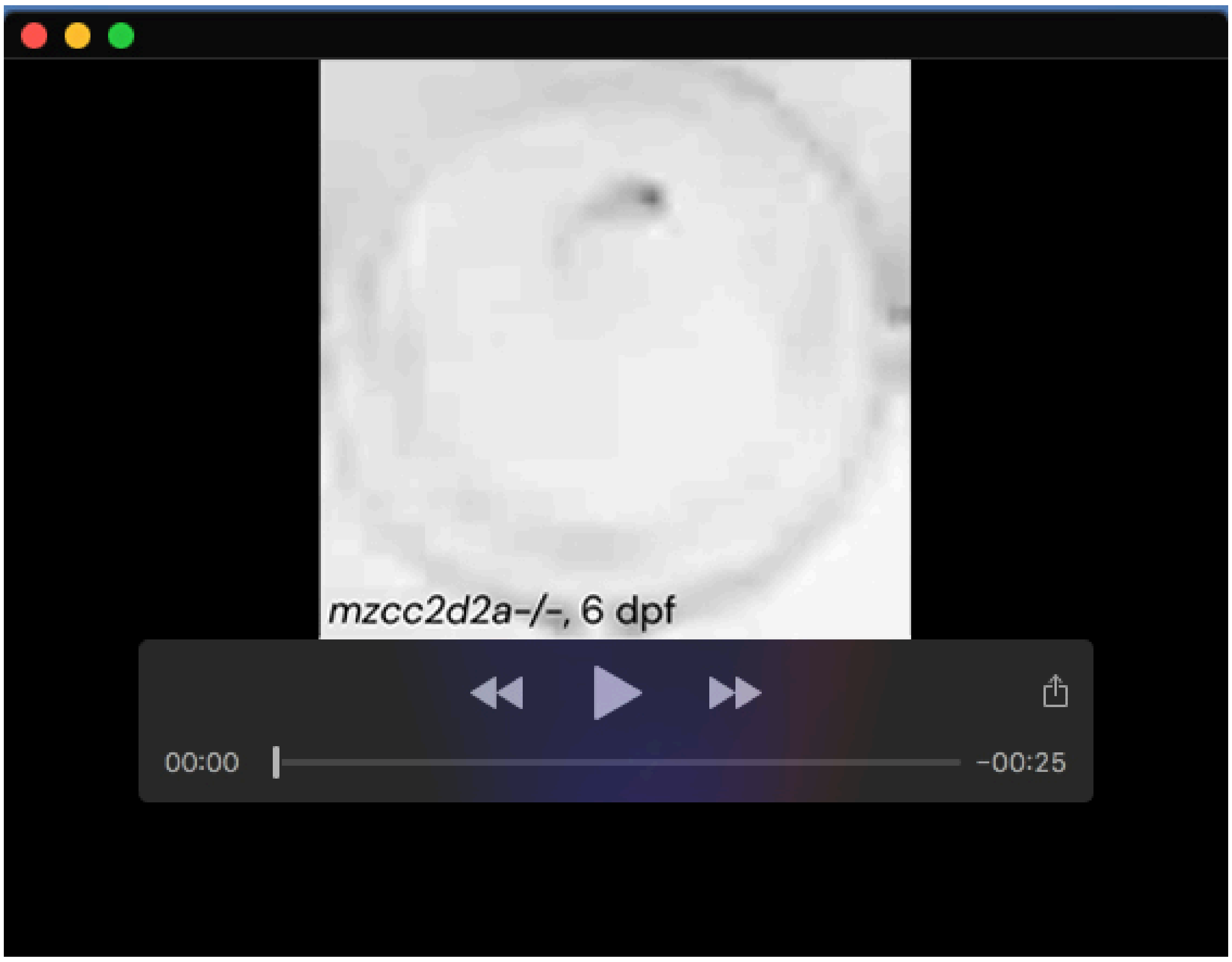

Movie 4. Swimming behaviour of a mz *cc2d2a* mutant larva at 6 dpf

Zebrabox recording of a mz *cc2d2a* mutant larva at 6 dpf, showing reduced swimming compared to control.

Biology Open • Supplementary information

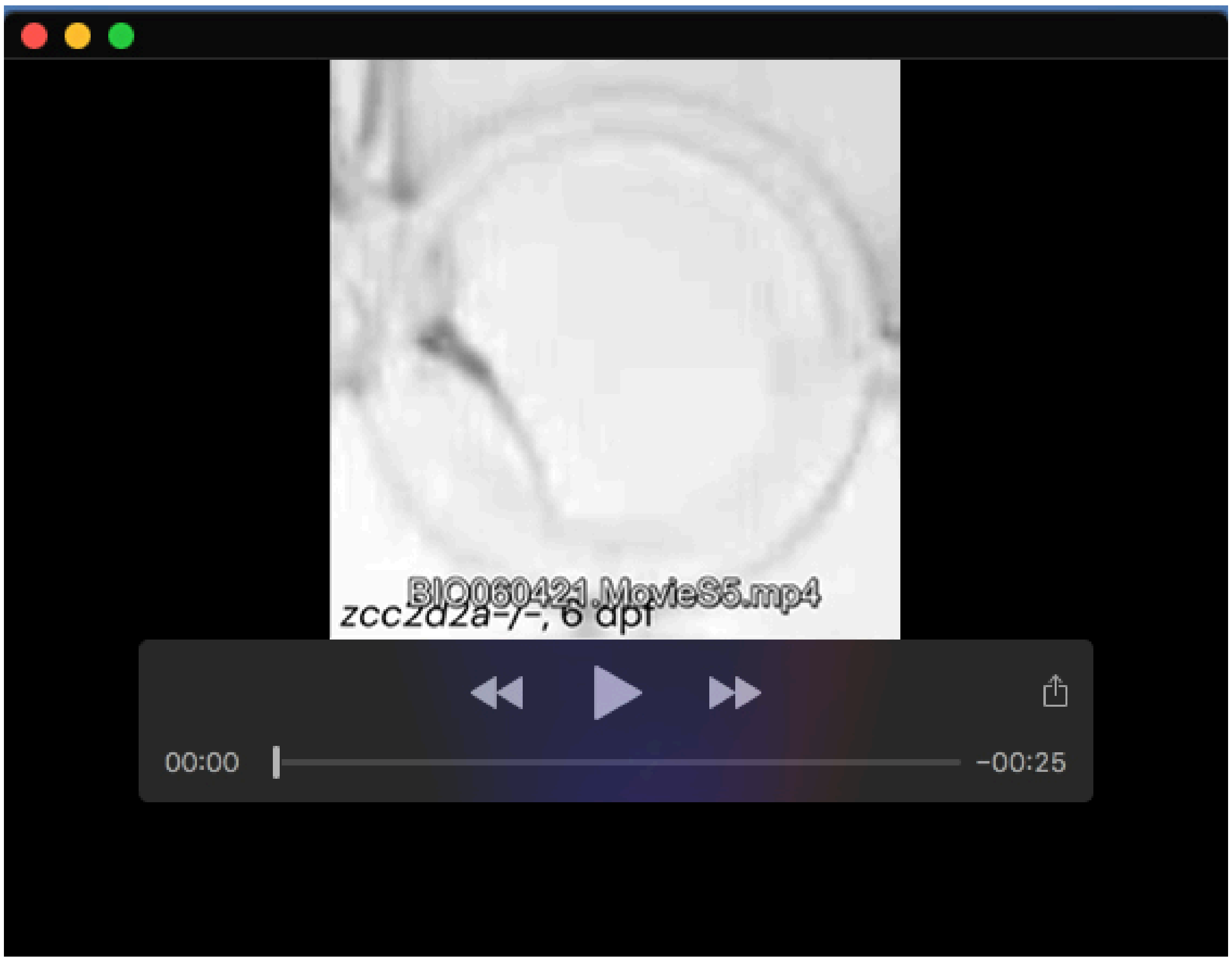

Movie 5. Swimming behaviour of a zygotic *cc2d2a* mutant larva at 6 dpf

Zebrabox recording of a zygotic *cc2d2a* mutant larva at 6 dpf, showing reduced swimming compared to control.

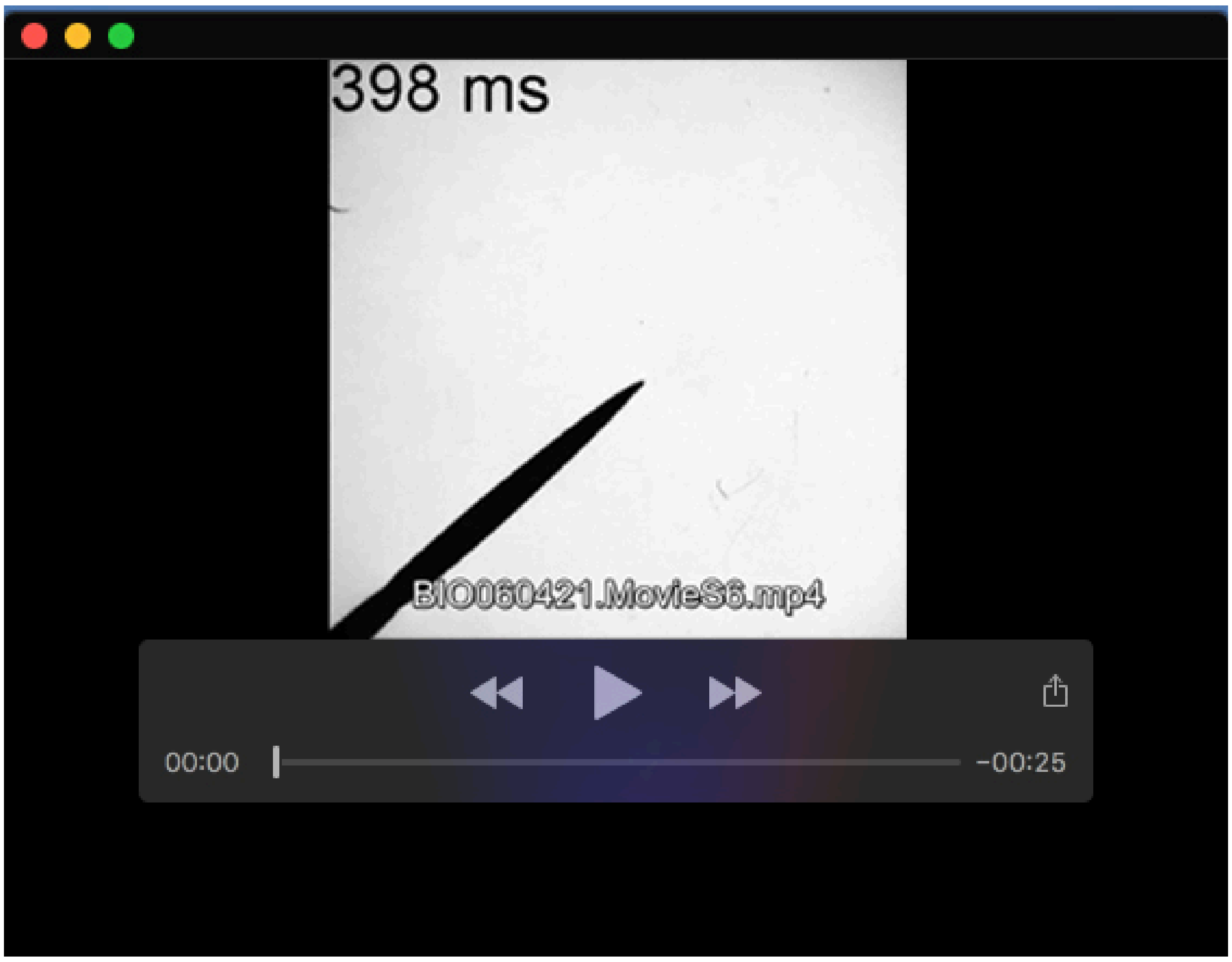

Movie 6. Touched-evoked escape response of a control larva at 2 dpf

Touch-evoked escape response of a control larva at 2 dpf, recorded using a widefield microscope. The larva exhibits a fast swimming response away from the touch stimulus. ms = milliseconds.

Biology Open • Supplementary information

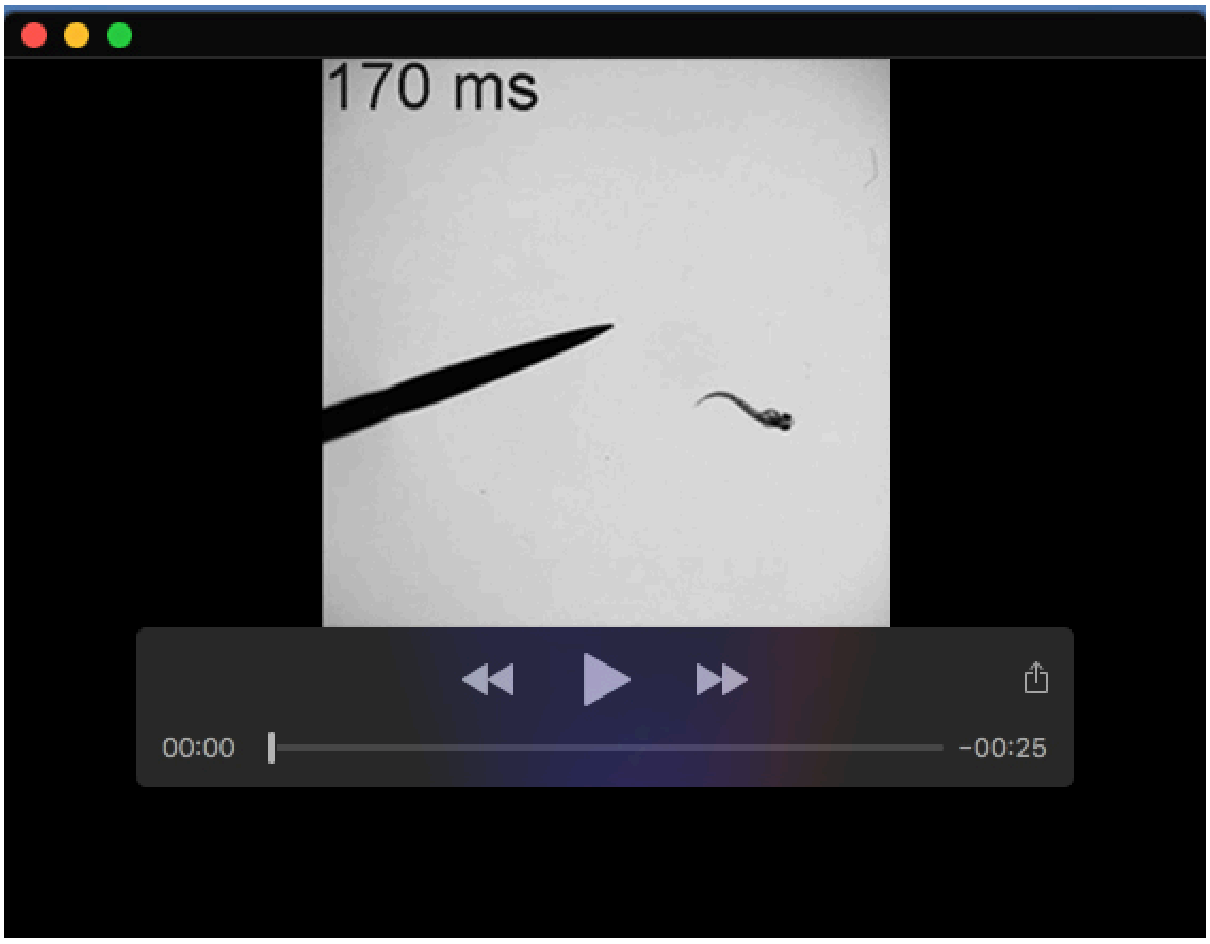

Movie 7. Touched-evoked escape response of a zygotic *cc2d2a* mutant larva at 2 dpf

Touch-evoked escape response of a zygotic *cc2d2a* mutant larva at 2 dpf, recorded using a widefield microscope. The larva exhibits an abnormal touch-evoked escape response compared to controls, swimming in a circular fashion and sometimes upside down. ms = milliseconds.

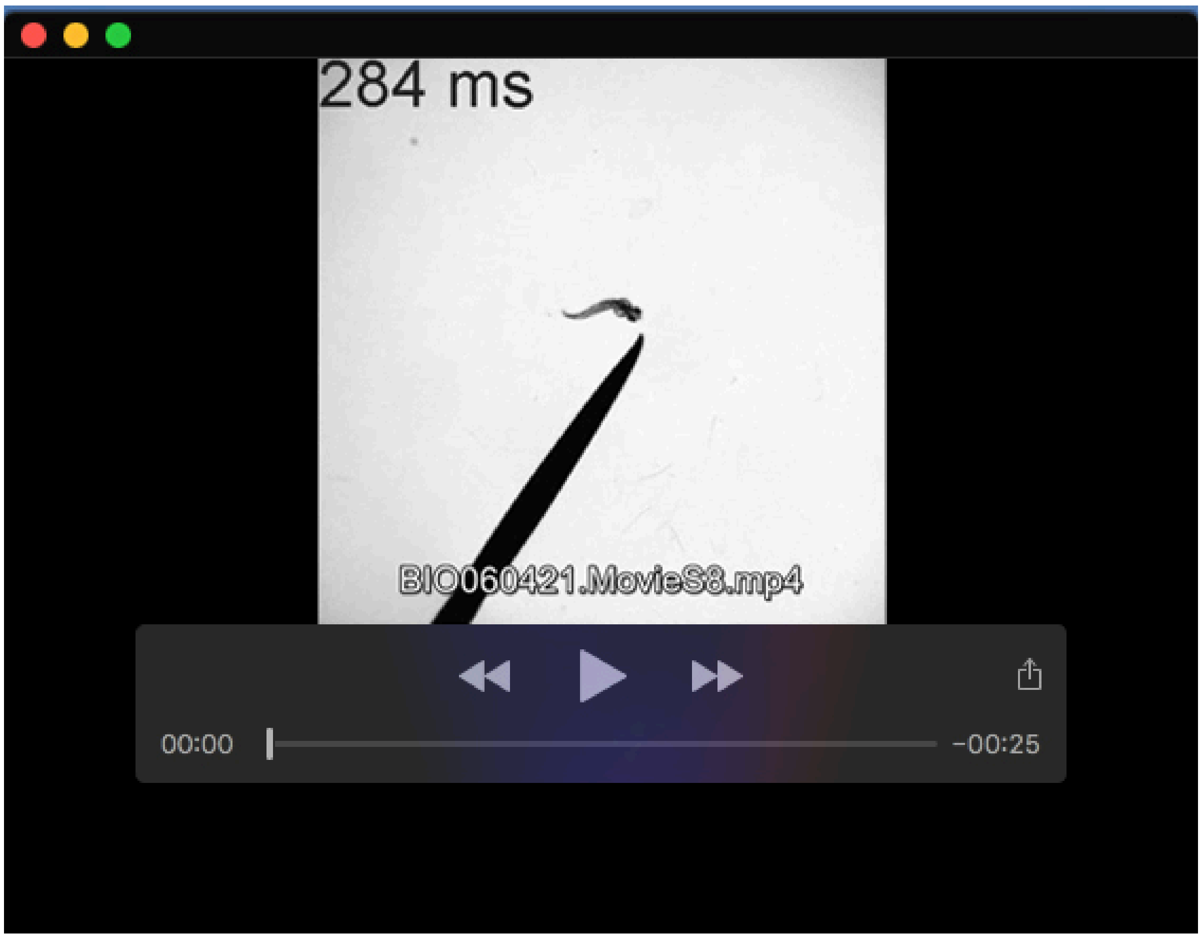

Movie 8. Touched-evoked escape response of a *talpid3* mutant larva at 2 dpf

Touch-evoked escape response of a zygotic *talpid3* mutant larva at 2 dpf, recorded using a widefield microscope. The touch-evoked escape response is comparable to controls. ms = milliseconds.

Biology Open • Supplementary information
